# Bacteria and Soil Enzymes Supporting the Valorization of Forested Soils

**DOI:** 10.3390/ma15093287

**Published:** 2022-05-04

**Authors:** Agata Borowik, Jadwiga Wyszkowska, Jan Kucharski

**Affiliations:** Department of Soil Science and Microbiology, University of Warmia and Mazury in Olsztyn, Plac Łódzki 3, 10-727 Olsztyn, Poland; agata.borowik@uwm.edu.pl (A.B.); jan.kucharski@uwm.edu.pl (J.K.)

**Keywords:** environmental catalysis, enzymes activity, forest soils, microbial communities

## Abstract

To decompose forest biomass, microorganisms use specific enzymes from the class of oxidoreductases and hydrolases, which are produced by bacteria and soil fungi. In post-agricultural forest soils, bacteria adapt more easily to changing ecological conditions than fungi. The unique features of bacteria, i.e., tolerance and the ability to degrade a wide range of chemical compounds, prompted us to conduct research that contributes to the improvement of the broadly understood circular management of biomass production and economic efficiency. This study aimed to analyze changes in the microbiological activity and the activities of dehydrogenases, catalase, β-glucosidase, urease, arylsulfatase, acid phosphatase, and alkaline phosphatase in the soil sampled from under *Picea abies* (Pa), *Pinus sylvestris* (Ps), *Larix decidua* (Ld), *Quercus robur* (Qr), and *Betula pendula* (Bp), after 19 years. The control object was unforested soil. The studies allowed one to demonstrate the relationship between the activity of soil enzymes and the assemblages of culturable microorganisms and bacteria determined by the metagenomic method and tree species. Thus, it is possible to design the selection of tree species catalyzing enzymatic processes in soil. The strongest growth promoter of microorganisms turned out to be *Quercus robur* L., followed by *Picea abies* L., whereas the weakest promoters appeared to be *Pinus sylvestris* L. and *Larix decidua* M.

## 1. Introduction

The exclusion of lands from agricultural production, and a change in soil management involving afforestation, has recently become a reversed trend of deforestation and land protection both in Europe and beyond [1,2]. The afforestation of weak soils prevents desertification, thereby minimizing the risk of soil erosion and contributing to the alleviation of climate changes [3]. A broad spectrum of the changes observed in the natural environment that are related to land use has recently spurred huge interest [1,4,5,6,7] as it affects the enzymatic and microbiological activity of soils [8,9]. Being the major element of the soil ecosystem, the soil microbiome is strongly related to the pedogenic processes responsible for soil formation. This aspect is often neglected and underestimated in research, while any modification in the composition of soil microorganisms affects organic matter degradation and the humification processes, and thus the accumulation of humus, which, thanks to its capacity to accumulate water as well as macro- and micro-nutrients, influences the contents of the nutrients available to plants [10]. Despite accounting for only a small part of the organic matter in the soil, microorganisms play a key role in its degradation and nutrient cycle [8].

According to Chao et al. [11], the concentration of CO_2_ in the air increases along with organic carbon degradation in the soil and plant biomass production. Potthoff et al. [12] found a higher content of CO_2_ in soils covered with vegetation than in black fallow soils. In turn, Wang et al. [10] claimed that drought is the factor that most significantly influences the carbon content of the soil because it reduces the absolute amount of carbon present in the soil, possibly due to a significant reduction in its photosynthetic fixation. Photosynthetic carbon is transferred to the soil via various routes, including by its allocation to mycorrhizal fungi [10]. The content of CO_2_ in the Earth’s atmosphere is growing rapidly and, according to many authors [13], it will increase in the future. In 2005–2010, the CO_2_ level increased by 3%, and in 2010–2020 by 6% (https://climate.nasa.gov/vital-signs/carbon-dioxide/, accessed on 21 February 2021) [14], while according to the Mouna Loa Observatory in Hawaii, its current level is at 0.0417% [14].

Soil plays a significant role in atmospheric CO_2_ sequestration. Carbon dioxide absorbs heat energy from the Earth’s surface, which causes the temperature to increase and climate changes to appear [13]. Forests are also capable of capturing and storing atmospheric CO_2_. The potentially important role of forest ecosystems in mitigating climate changes suggests a strong need for their more detailed research [15].

The research by Smal et al. [2] has shown that afforestation of post-agricultural soils allowed for their transformation into typical forest soils. However, it remains unknown whether the changes in soil properties, including changes in enzymatic activity, follow the same direction and steady pace, as well as how long it takes to develop the conditions characteristic for a stabilized forest ecosystem. According to Dhillion et al. [16], the amount of carbon pervading the soil in the form of CO_2_ is largely determined by the activity of microorganisms. Increased CO_2_ content stimulates organic matter degradation and leads to the regression or release of nutrients in the soil [17,18]. The microbial biomass of soil is an important ecological indicator of its fertility and quality [19,20]. Changes in the microbial populations in response to increased CO_2_ levels were observed by Drigo et al. [21]. They afflicted separate groups of opportunistic microorganisms closely related to plants. Changes in the number and diversity of microorganisms in the rhizosphere have been found to depend on the species of plants present; the stage of their development; the morphology of their root system; pH; and the composition of the released chemicals [22,23]. According to Uksa et al. [24], young plant roots stimulate microbial growth more actively due to the intense dynamics of the physiological and biochemical processes in their cells. In contrast, the roots of mature and aging plants release more complex carbon compounds and/or secondary metabolites, thereby enriching more specialized microbial communities [25,26]. The impact of plants on the communities of microorganisms is related to the activity of soil enzymes [27,28], which is difficult to interpret due to the lack of threshold values defined for various ecosystems [29,30]. Nevertheless, enzyme activities reflect the processes ongoing in the soil. Therefore, understanding soil enzyme responses to land-use changes is critical to soil health and to increasing its productivity [27,31]. According to Pandey et al. [32] and de Medeiros et al. [33], enzymatic activity is considered to be one of the most sensitive indicators of soil fertility and productivity, as well as of soil microorganism diversity. Our research hypothesized that the microbiological and enzymatic properties change depending on the type of land use/land cover and that the species composition of trees used for afforestation has a significant impact on the composition of the soil bacterial communities and the activity of enzymes involved in the carbon, nitrogen, phosphorus, and sulfur cycles. This hypothesis was verified in studies aimed to determine the diversity of bacteria at various taxonomic levels and the activities of dehydrogenases, catalase, β-glucosidase, urease, acid and alkaline phosphatase, and arylsulfatase in the soil afforested with *Picea abies* L., *Pinus sylvestris* L., *Larix decidua* M., *Quercus robulaur* L., and *Betula pendula* L.

## 2. Materials and Methods

### 2.1. Characteristics of the Study Area

**Figure 1:**
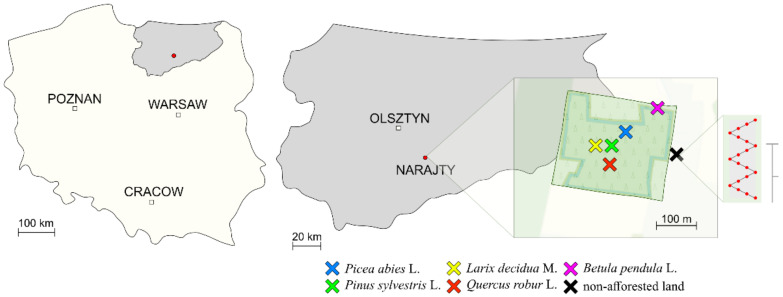
A map of the soil sampling sites. The Narajty in the Pasym commune (53°36′00.8″ N 20°47′25.1″ E) of the Warmian–Masurian Voivodeship (Poland). 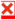
—15,900 seedlings of English oak (*Quercus robur* L.—Qr) planted on the area of 1.95 ha; 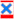
—4500 seedlings of Norway spruce (*Picea abies* L.—Pa) planted on the area of 0.97 ha; 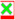
—7700 seedlings of Scots pine (*Pinus sylvestris* L.—Ps) planted on the area of 0.97 ha; 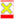
—700 seedlings of European larch (*Larix decidua* M.—Ld) planted on the area of 0.43 ha; 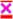
—2400 seedlings of warty birch (*Betula pendula* L.—Bp) planted on the area of 0.55 ha; and 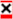
—non-afforested land. 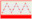
—a soil sampling scheme with the use of Egners Riehm’s staff.

The research was carried out in 2020 and aimed to analyze the microbiological and biochemical properties of the soil from the post-agricultural forest located in Narajty in the Pasym commune (53°36′00.8″ N 20°47′25.1″ E) of the Warmian–Masurian Voivodeship (Poland) (Figure 1).

According to the physico-geographical division, the study area is located in the Olsztyn Lakeland (842.81), which is part of the Masurian Lakeland (842.8), which is included in the East European Plain [34]. The landscape of the Olsztyn Lakeland was shaped as a result of the Pleistocene glaciation. The area has a warm temperate transition climate. The average annual air temperature in 2018 was +9.0 °C and was 1.1 °C higher than the average value for the twenty-year period of 1998–2018 [35]. The annual sum of precipitation reached 550 mm and was 16% lower than the average for the two decades (1998–2018).

The forest that was the subject of the research was established in 2006 on 4.87 ha of arable land with a grain size composition of loamy sand (Table S1). It was established using the following tree species: 15,900 seedlings of English oak (*Quercus robur* L.—Qr) planted on the area of 1.95 ha; 4500 seedlings of Norway spruce (*Picea abies* L.—Pa) planted on the area of 0.97 ha; 7700 seedlings of Scots pine (*Pinus sylvestris* L.—Ps) planted on the area of 0.97 ha; 700 seedlings of European larch (*Larix decidua* M.—Ld) planted on the area of 0.43 ha; and 2400 seedlings of warty birch (*Betula pendula* L.—Bp) planted on the area of 0.55 ha. The seedlings of *Picea abies* L., *Pinus sylvestris* L., and *Larix decidua* M. were planted between the oak seedlings. Each of these species was planted in three clumps. The area afforested with *Quercus robur* L., *Picea abies* L., *Pinus sylvestris* L., and *Larix decidua* M. was surrounded by *Betula pendula* L., which was planted in four rows around the plot. Three rectangular-shaped plots (20 m × 5 m) were marked out in the non-afforested area surrounding the forest and in the area that was afforested with the individual tree species, which gave 18 plots in total. From each plot, 20 single soil samples were collected from a depth of 0–20 cm and combined into 18 bulk samples (3 plots × 6 test objects). The individual soil samples were collected with an Egners Riehm’s staff.

### 2.2. Chemical and Physicochemical Analyses of Soil

For physicochemical analyses, the soil was dried at a temperature of 60 °C to the air-dry weight and sieved through a screen with a mesh diameter of 2 mm. The soil samples were determined for grain size composition, using a laser meter; organic carbon content, with the Tiurin method [36]; total nitrogen content, with the Kjeldahl method [37]; the contents of available phosphorus and potassium, with the Egner et al. [38] method; magnesium content, using atomic absorption spectrometry [39]; the contents of exchangeable K^+^, Ca^2+^, Mg^2+^, and Na^+^ cations, according to ISO 11,260 standard [40]; pH value, with the potentiometric method [41]; and hydrolytic acidity (HAC), the content of exchangeable base cations (EBC), and cation exchange capacity (CEC), according to Carter and Gregorich [42]. Chemical and physicochemical properties were determined with the following equipment: Malvern Mastersizer 2000 Laser Diffraction (Malvern, Worcestershire, UK), Spectrophotometer SQ118 (Merc, Darmstadt, Germany), Buchi B-324 distiller (Buchi, Flawil, Switzerland), Jenway 6705 UV/VIS spectrophotometer (Jenway LTD, Staffordshire, UK), Jenway PFP 7 flame photometer (Jenway LTD, Staffordshire, UK), and atomic absorption spectrophotometer GBC 932AA (GBC Scientific Equipment, Braeside, Australia). The results of these determinations are presented in Tables S2 and S3.

### 2.3. Microbiological and Enzymatic Analyses of Soil

The serial dilution method was employed to determine counts of copiotrophic bacteria (Cop), actinobacteria (Act), and fungi (Fun) in the soil samples, using culture media characterized in the work by [29]. The Cop, Act, and Fun colonies were used to determine the colony development index (CD) and the ecophysiological diversity index (EP) of these microorganisms. The number of colony-forming units (cfu) was established using a colony counter. Microbial counts determined for 10 subsequent days were used to compute the colony development index (CD) and the ecophysiological diversity index (EP) of microorganisms, according to De Leij et al. [43] based on the following formulas:

CD = [N1/1 + N2/2 + N3/3… N10/10] · 100(1)

where N1, N2, N3, …, N10 are the sum of ratios of the number of colonies of microorganisms identified in particular days (1, 2, 3, …, 10) to the total number of colonies identified throughout the study period, and

EP = −Σ(pi·log10 pi)(2)

where pi represents the ratio of the number of colonies of microorganisms identified in particular days to the total number of colonies identified throughout the study period.

The colony development index (CD) indicates the rate of succession of microorganisms in the environment, while the ecophysiological diversity index (EP) of microorganisms indicates the ecophysiological diversity of microorganisms determined by changing environmental conditions. The detailed procedures used to determine counts of microorganisms and calculate values of CD and EP indices were described in our previous work [44].

**Figure 2:**
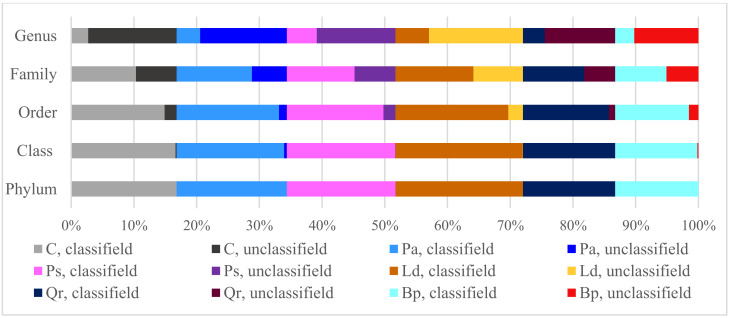
The total number of OTU. C—non-afforested land, Pa—*Picea abies* L., Ps—*Pinus sylvestris* L., Ld—*Larix decidua* M., Qr—*Quercus robur* L., and Bp—*Betula pendula* L.

Furthermore, DNA was isolated from soil samples using the Genomic Mini AX Bacteria+ kit (A&A Biotechnology). The 1055F (5′-ATGGCTGTCGTCAGCT-3′) and 1392R (5′-ACGGGCGGTGTGTAC-3′) primers were used in the PCR reaction mixture, whereas the 341F and 785R primers were used for the amplification of the hypervariable V3-V4 region of the 16S rRNA gene. New-generation sequencing was conducted on the Ilummina MiSeq v2 platform (Illumina Inc., San Diego, CA, USA) in the paired-end (PE) technology, 2 × 250 pz. The bioinformatic analysis was carried out using QIIME packages based on reference databases of sequences GreenGenes v13_8 (Genomed S.A., Warsaw, Poland). Sequencing data have been deposited with GenBank NCBI https://www.ncbi.nlm.nih.gov/ (accessed on 21 February 2021) under accession numbers MW601841-MW601938. The resulting sequences were grouped into operational taxonomic units (OTU), which were employed to determine the diversity of bacteria using the Shannon–Wiener index (H’) [45]. The exact procedure of the metagenomic analyses was provided in our earlier work [43]. The accuracy of the taxonomic classification of bacteria varied. On average, regardless of the study object, 100% of the OTU were classified to the phylum, 99% to the class, 90% to the order, 63% to the family, and barely 23% to the genus (Figure 2).

The soil samples were also analyzed for enzymatic activity, including the activities of dehydrogenases, according to Öhlinger [46]; catalase, according to Johnson and Temple [47]; and urease, acid phosphatase, alkaline phosphatase, β-glucosidase, and arylsulfatase, according to Alef and Nannpieri [48]. The activity of dehydrogenases was expressed in the mMol triphenyl formazan (TFF); that of catalase in Mol O_2_; that of urease in mMol N-NH_4_^+^; and those of acid phosphatase, alkaline phosphatase, β-glucosidase, and arylsulfatase in mMol 4-nitrophenol (PN) per 1 kg of soil dry matter per 1 h.

### 2.4. Statistical Analysis

The statistical analysis of results was carried out using the Statistica 13.3 package TIBCO Software Inc. [49]. The normality of distribution was checked with the Shapiro–Wilk test [50] and the Kruskal–Wallis test [51]. The results featuring normal distribution were then analyzed with the Duncan post-hoc test, whereas those not featuring normal distribution were compared using the Dunn post-hoc test in Bonferroni’s modification.

The counts of microorganisms and the activities of soil enzymes were verified in the principal component analysis (PCA). Additional computations were made for the index of a tree stand effect (IF_T_) on the microbiological and enzymatic properties of the soil:

(3) $${\mathrm{IF}}_{T}=\frac{A_{T}}{A_{0}}-1$$

where

IF_T_—the index of a tree stand effect;
A_T_—the count of microorganisms/the activity of enzymes in the soil from the afforested area;
A_0_—the count of microorganisms/the activity of enzymes in the soil from the non-afforested area.

A negative value of IF_T_ indicates an adverse effect, IF_T_ = 0 indicates no effect, and a positive value of IF_T_ indicates a positive effect, of afforestation.

The metagenomic data obtained were presented graphically using the STAMP 2.1.3. software [52]. Data in the circular arrangement were analyzed using the Circos 0.68 package [53], where the presented OTU values are directly proportional to the width of each band linking bacterial taxa with the characterized object. Different colors are assigned to each bacterial family. The external ring represents the total percentage of 16S sequences, whereas the internal ring represents the number of 16S amplicon sequences assigned to a given taxon. A heat map characterizing bacterial genera was plotted using the RStudio v1.2.5033 software (RStudio 2019), the R v3.6.2 system (R Core 2019), and the gplots library [54]. The Venn diagram was plotted using a tool for the analysis of sets [55]. All graphical data are presented after eliminating OTU smaller than 1% of the total OTU number.

## 3. Results

**Figure 3:**
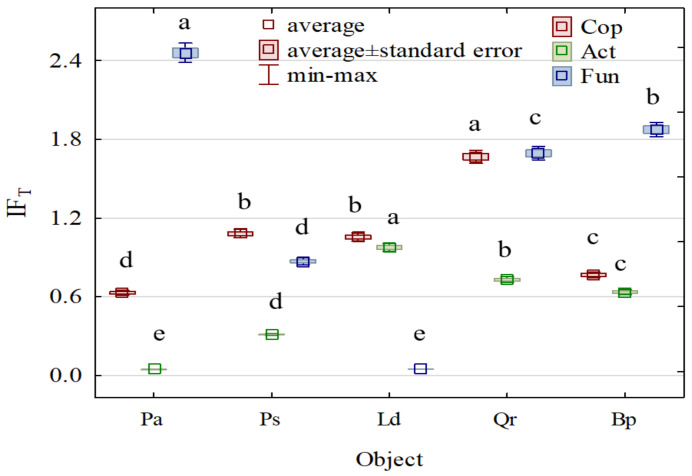
The index of the stand influence on the number of microorganisms. Pa—*Picea abies* L., Ps—*Pinus sylvestris* L., Ld—*Larix decidua* M., Qr—*Quercus robur* L., Bp—*Betula pendula* L., Cop—copiotrophic bacteria, Act—actinomycetes, and Fun—fungi. Homogeneous groups (denoted with letters a–e) were calculated separately for each microorganism.

Soil afforestation with various tree species had a positive effect on the proliferation of soil microorganisms (Figure 3), although it obviously limited the counts of microorganisms to a different extent. Nevertheless, regardless of tree species, the strongest tree stand’s effect was noted on the number of fungi, followed by the number of copiotrophic bacteria, whereas the weakest one was noted on the number of actinobacteria. The individual tree species caused the most significant differences in the population of soil fungi. *Picea abies* L. caused the greatest increase in their number (IF_T_ = 2.460), followed by *Betula pendula* L. (IF_T_ = 1.875) and *Quercus robur* L. (IF_T_ = 1.694), whereas *Larix decidua* M. caused the smallest increase in their population (IF_T_ = 0.049). In contrast to fungi, the weakest effect of afforestation on copiotrophic bacteria and actinobacteria was attributable to *Picea abies* L. (IF_T_ = 0.630 and IF_T_ = 0.046, respectively). The most intensive proliferation of copiotrophic bacteria was observed in the soil sampled from the areas afforested with *Quercus robur* L. (IF_T_ = 1.668), *Pinus sylvestris* L. (IF_T_ = 1.081), and *Larix decidua* M. (1.055), whereas that of actinobacteria was observed in the soil sampled from the areas afforested with *Larix decidua* M. (IF_T_ = 0.977), *Quercus robur* L. (IF_T_ = 0.730), and *Betula pendula* L. (IF_T_ = 0.635).

**Figure 4:**
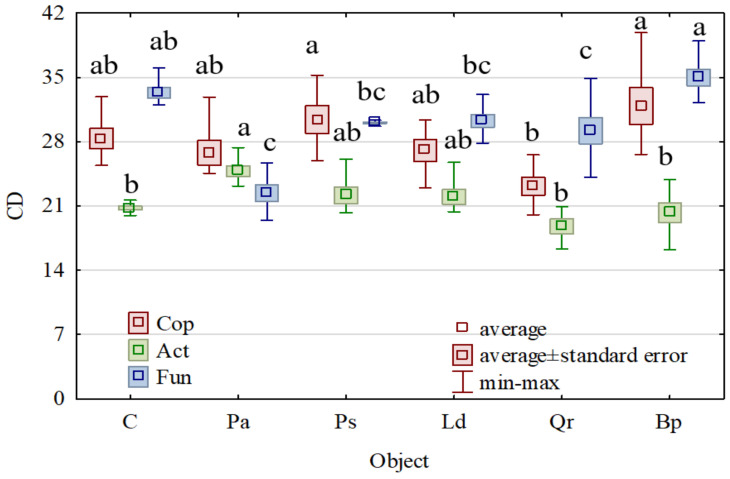
The colony development index (CD) for Cop—copiotrophic bacteria, Act—actinomycetes and Fun—fungi. C—non-afforested land, Pa—*Picea abies* L., Ps—*Pinus sylvestris* L., Ld—*Larix decidua* M., Qr—*Quercus robur* L., and Bp—*Betula pendula* L. Homogeneous groups (denoted with letters a–c) were calculated separately for each microorganism.

The afforestation caused less significant changes in the colony development index (CD) values than in the counts of the microorganisms (Figure 4). European oak triggered the most significant changes in the CD values of copiotrophic bacteria and actinobacteria, whereas *Picea abies* L. did so in the CD value of fungi. These CD values were significantly lower than the respective CD values obtained for the soil sampled from the non-afforested area, indicating that these tree species modified the structure of copiotrophic bacteria, actinobacteria, and fungi from the k strategists to the R strategists.

**Figure 5:**
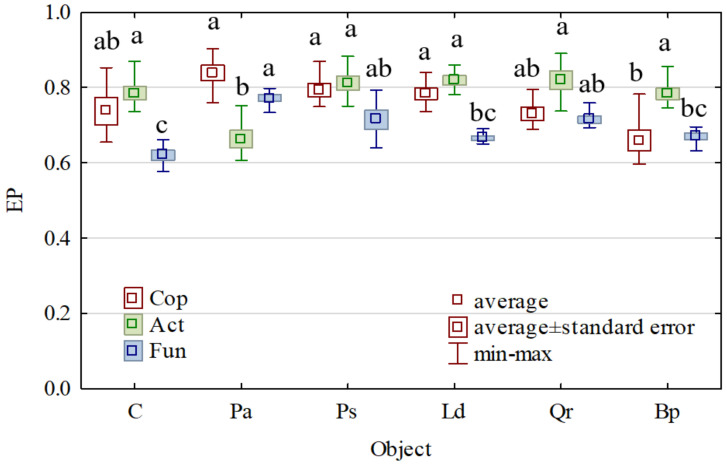
The ecophysiological diversity factor (EP) for Cop—copiotrophic bacteria, Act—actinomycetes and Fun—fungi. C—non-afforested land, Pa—*Picea abies* L., Ps—*Pinus sylvestris* L., Ld—*Larix decidua* M., Qr—*Quercus robur* L., and Bp—*Betula pendula* L. Homogeneous groups (denoted with letters a–c) were calculated separately for each microorganism.

Not all tree species had the same effect on the ecophysiological diversity index (EP) of the microorganisms tested (Figure 5). The highest EP values of copiotrophic bacteria and fungi were noted in the soil afforested with *Picea abies* L. (EP = 0.839 and EP = 0.772, respectively). In the case of actinobacteria, the highest EP values were caused by soil afforestation with *Pinus sylvestris* L. (EP = 0.810), *Larix decidua* M. (EP = 0.819), and *Quercus robur* L. (EP = 0.820), whereas the lowest ones were caused by soil planting with *Picea abies* L. (EP = 0.663).

**Figure 6:**
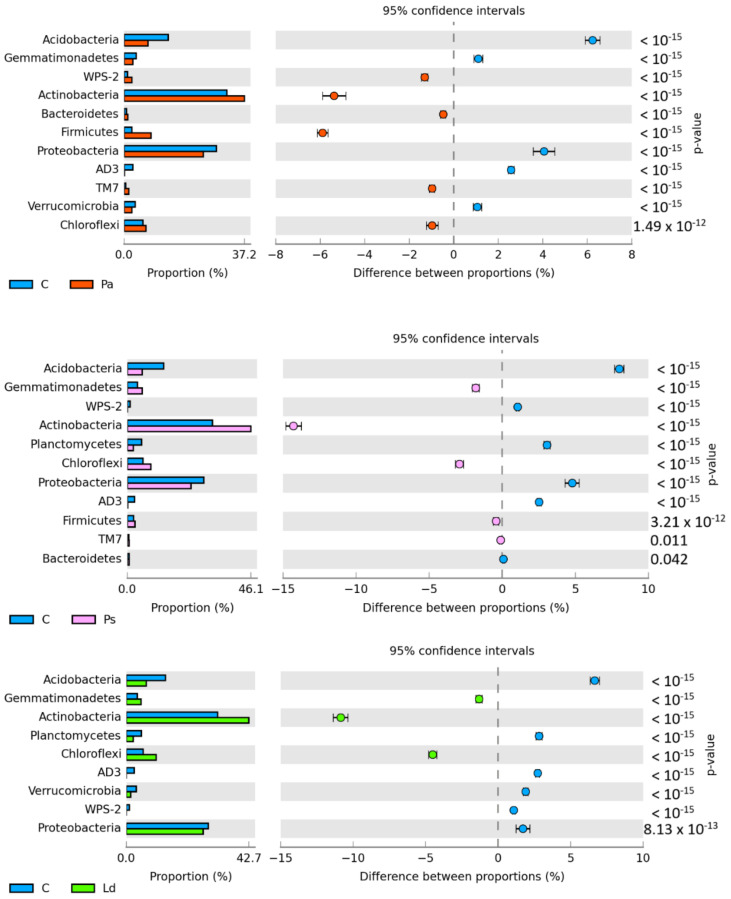

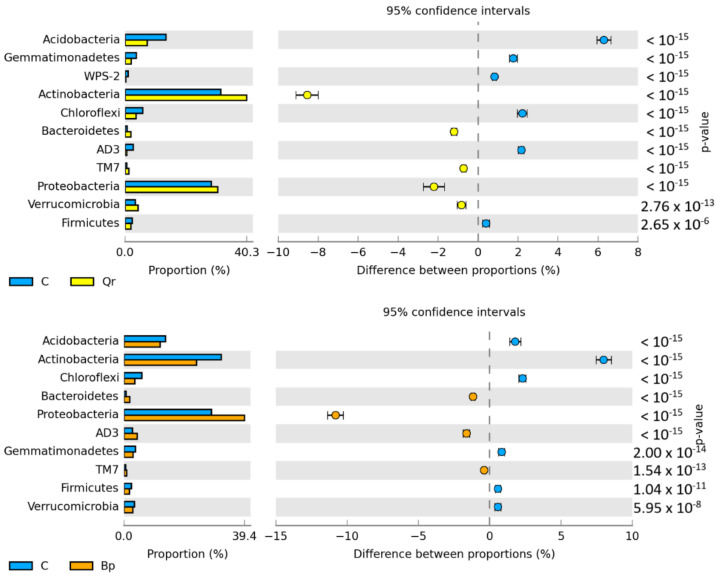
The relative abundance of the dominant phylum bacteria in forest soils with the difference between the proportions ≥1%. C—non-afforested land, Pa—*Picea abies* L., Ps—*Pinus sylvestris* L., Ld—*Larix decidua* M., Qr—*Quercus robur* L., and Bp—*Betula pendula* L.

The OTU number of bacteria in the soil depended on tree species used for afforestation. It ranged from 50,705 OTU in the soil afforested with *Betula pendula* L. to 76,682 OTU in the soil afforested with *Larix decidua* M. *Picea abies* L. and *Pinus sylvestris* L. caused minor changes in the relative OTU number, whereas *Larix decidua* M. increased it by 21%, while *Quercus robur* L. and *Betula pendula* L. decreased it by 13% and 21%, respectively, compared to the relative OTU number determined in the control soil. The predominant phylum in the control soil and in all soils from the afforested areas, except for the area planted with *Betula pendula* L., turned out to be *Actinobacteria* (Figure 6). Their contribution to the OTU structure ranged from 31.8% in the control soil to 46.1% in the soil afforested with *Pinus sylvestris* L. In the soils afforested with *Picea abies* L., *Larix decidua* M., and *Quercus robur* L., it accounted for 37.2%, 42.7%, and 40.3%, respectively. The greatest contribution of *Proteobacteria* (39.4%) and a significantly lower one of *Actinobacteria* (23.8%) were found only in the soil afforested with *Betula pendula* L. In the remaining plots, *Proteobacteria* ranked second after *Actinobacteria*; however, the relative OTU number of this phylum was less diversified among the plots and ranged from 23.8% in the soil afforested with *Pinus sylvestris* L. to 30.8% in the control soil and soil afforested with *Quercus robur* L.

**Figure 7:**
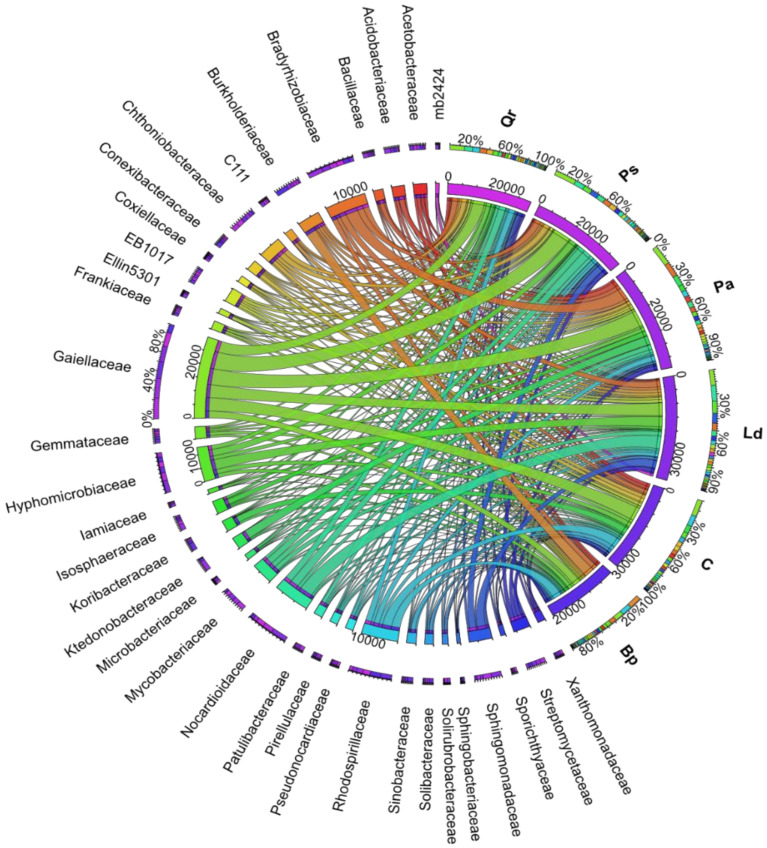
The relative abundance of the dominant families’ bacteria in forest soils with the difference between the proportions ≥1%. The operational taxonomic unit (OTU) values of families’ bacteria provide data in direct proportion to the width of each band connecting the bacterial taxa to the respective soil sample. A specific color is assigned to each family of bacteria. The inner ring represents the number of 16S amplicon sequences assigned to a given taxon, while the outer ring represents the total percentage of 16S sequences. C—non-afforested land, Pa—*Picea abies* L., Ps—*Pinus sylvestris* L., Ld—*Larix decidua* M., Qr—*Quercus robur* L., and Bp—*Betula pendula* L.

On average, regardless of the plot, the highest numbers of OTU were assigned to the *Gaiellaceae, Bradyrhizobiaceae, Rhodospirillaceae, Hyphomicrobiaceae*, and *Nocardioidaceae* families (Figure 7). Nevertheless, the tree stands modified the family structure. Thus, the *Gaiellaceae, Bradyrhizobiaceae*, and *Ktedonobacteraceae* families prevailed in the soil afforested with *Picea abies* L.; the *Gaiellaceae, Nocardioidaceae*, and *Hyphomicrobiaceae* families, in the soil planted with *Pinus sylvestris* L.; the *Gaiellaceae, Nocardioidaceae*, and *Hyphomicrobiaceae* families, in the soil afforested with *Larix decidua* M.; the *Gaiellaceae*, *Nocardioidaceae*, and *Rhodospirillaceae* families, in the soil afforested with *Quercus robur* L.; and the *Burkholderiaceae, Rhodospirillaceae*, and *Gaiellaceae* families, in the soil planted with *Betula pendula* L.

**Figure 8:**
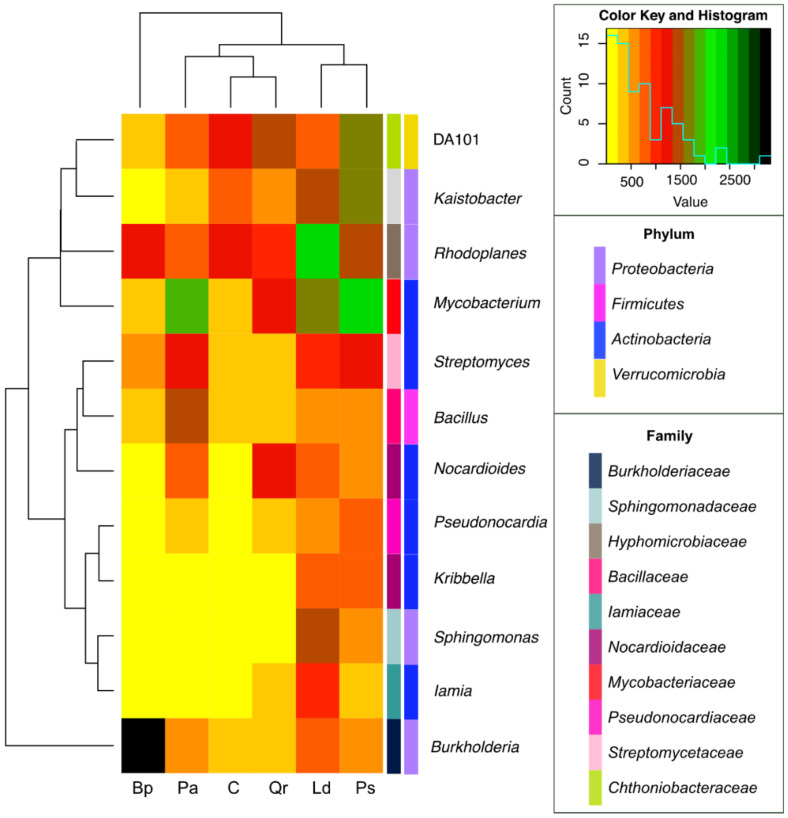
A heat map presenting associations between bacterial genera in the forest soils tested with the difference between ratios ≥1%. The smallest amounts of bacterial OTU are shown in yellow, while the largest amounts of OTU are shown in black. The blue line shown in the “color key and histogram” shows how many times each data point appears in the matrix used in the heat map.C—non-afforested land, Pa—*Picea abies* L., Ps—*Pinus sylvestris* L., Ld—*Larix decidua* M., Qr—*Quercus robur* L., and Bp—*Betula pendula* L.

All tree species significantly decreased the number of OTU assigned to the *Koribacteraceae, Sinobacteraceae, Conexibacteraceae, Solibacteraceae, Gemmataceae*, and *Acetobacteraceae* families, whereas they increased the number of OTU classified to the *Bradyrhizobiaceae, Burkholderiaceae*, and *Mycobacteriaceae* families. Among all tree stands, *Picea abies* L. definitely had the strongest effect on the abundance of OTU from the family *Bradyrhizobiaceae* (an increase by 7.5% compared to the control soil). In turn, *Pinus sylvestris* L. (increase by 6.5%), *Larix decidua* M. (increase by 1.5%), and *Quercus robur* L. (increase by 6.2%) had the strongest impact on *Nocardioidaceae* OTU abundance, whereas *Betula pendula* L. did on *Burkholderiaceae* OTU abundance (increase by 13.2%). Six bacterial genera, i.e., *Kribbella* and *Nocardioides* (f_*Nocardioidaceae), Iamia* (f_*Iamiaceae), Mycobacterium* (f_*Mycobacteriaceae), Pseudonocardia* (f_*Pseudonocardiaceae)*, and *Streptomyces* (f_*Streptomycetaceae)*, were classified in the phylum *Actinobacteria*; four genera, including *Kaistobacter* and *Sphingomonas* (f_*Sphingomonadaceae), Burkholderia* (f_*Burkholderiaceae)*, and *Rhodoplanes* (f_*Hyphomicrobiaceae*), were classified in the phylum *Proteobacteria*; whereas *DA101* genus (f_*Chthoniobacteraceae*) was classified in the phylum *Verrucomicrobia* and *Bacillus* genus (f_*Bacillaceae*) in the phylum *Firmicutes* (Figure 8). Regardless of the plot, *Rhodoplanes* (16,0%) made the greatest contribution to the genus structure, followed by *Mycobacterium* (14.0%), *Burkholderia* (13.4%), and *DA101* (12.8%). The contribution of the other identified genera was below 10.0% and ranged from 2.6% for *Iamia* to 8.7% for *Kaistobacter*.

Regardless of the bacterial genus, the afforestation of arable soil contributed to an increase in the OTU number. Compared to the control soil, the OTU number increased 1.37-fold in the soil afforested with *Pinus sylvestris* L., 1.35-fold in the soil planted with *Larix decidua* M., 0.54-fold in the soil afforested with *Picea abies* L., 0.35-fold in the soil afforested with *Quercus robur* L., and 0.25-fold in that afforested with *Betula pendula* L. All tree species increased the OTU numbers of the *Sphingomonas* and *Mycobacterium* genera, though to a different extent. *Larix decidua* M., *Pinus sylvestris* L., *Quercus robur* L., and *Picea abies* L. caused a 63.23-fold, 60.15-fold, 13.00-fold, and 9.69-fold increase in OTU number of the genus *Kribbella*. This bacterial genus revealed the strongest response to the afforestation. It was followed by the genus *Sphingomonas*, and then by the *Iamia, Mycobacterium, Nocardioides, Streptomyces*, and *Burkholderia* genera.

**Figure 9:**
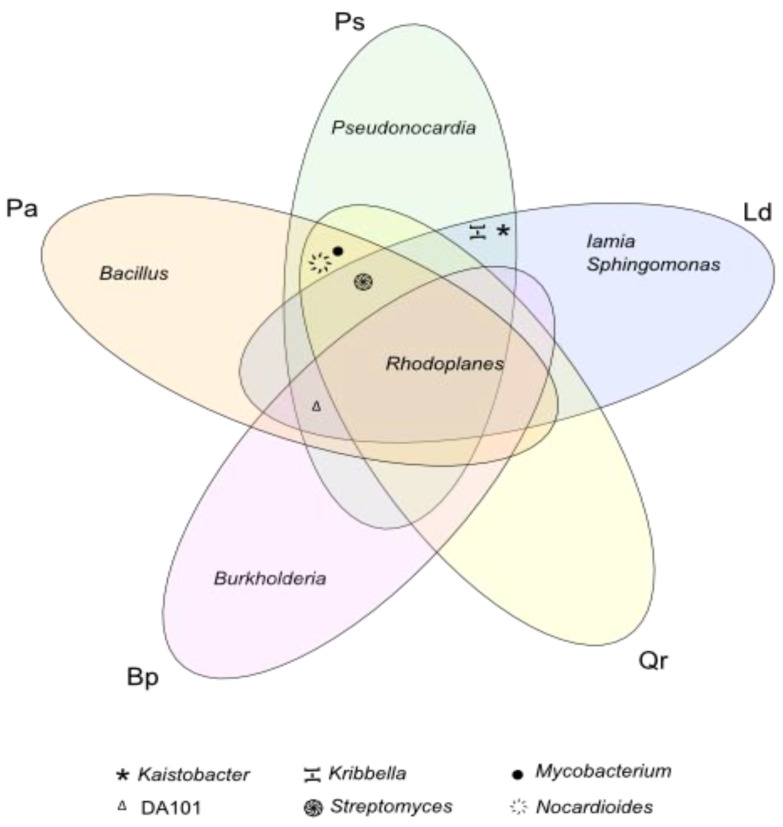
A Venn diagram showing common and unique species of bacteria in forest soils with the difference between the proportions ≥1%. Pa—*Picea abies* L., Ps—*Pinus sylvestris* L., Ld—*Larix decidua* M., Qr—*Quercus robur* L., and Bp—*Betula pendula* L.

Considering the above data, it may be concluded that the greatest diversity of bacteria at the class and order level occurred in non-afforested soil and from *Betula pendula* L.; at the family level, in soil near *Quercus robur* L.; and at the level of the genus, in *Pinus sylvestris* L. and *Larix decidua* M. (Table 1). *Bacillus* was the most typical of the soil afforested with *Picea abies* L.; the genus *Pseudonocardia*, of the soil planted with *Pinus sylvestris* L.; the *Sphingomonas* and *Iamia* genera, of the soil sampled from the *Larix decidua* M. stand; and the genus *Burkholderia*, of the soil afforested with *Betula pendula* L. (Figure 9).

**Figure 10:**
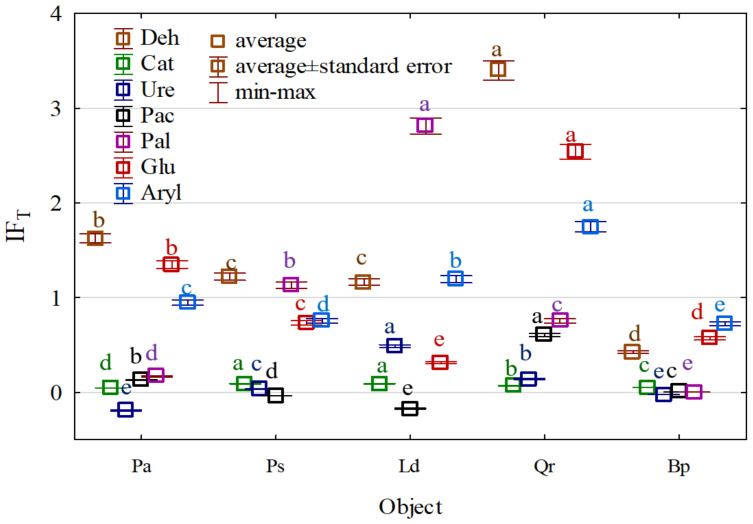
The index of forest stand influence on enzyme activity. Deh—dehydrogenases; Ure—urease, Pal—alkaline phosphatase, Pac—acid phosphatase, Aryl—arylsulphatase, Glu—*β*-glucosidase. Pa—*Picea abies* L., Ps—*Pinus sylvestris* L., Ld—*Larix decidua* M., Qr—*Quercus robur* L., and Bp—*Betula pendula* L. Homogeneous groups (denoted with letters a–e) were calculated separately for each enzyme.

The enzymatic activity of the soil sampled from all tree stands was highly diversified (Figure 10 and Table S4). All tree species stimulated the activities of dehydrogenases, β-glucosidase, and arylsulfatase. In turn, they had the weakest stimulating effect on the activities of catalase, urease, and acid phosphatase. The activity of alkaline phosphatase was positively affected by all tree species, except for *Betula pendula* L. Despite various fluctuations, data presented in Figure 8 indicate that *Quercus robur* L. and *Larix decidua* M. were the strongest promoters of the soil’s enzymatic activity, whereas *Betula pendula* L. was the poorest one.

## 4. Discussion

Interactions between vegetation, soil conditions, and climate strongly influence soil microorganisms [8,22,23,26,56]. In the present research, soil afforestation with various tree species promoted the multiplication of these microorganisms. Excluding lands from agricultural production and the afforestation of soils with *Picea abies* L., *Pinus sylvestris* L., *Larix decidua* M., and *Quercus robur* L. i *Betula pendula* L. reduced the number of microorganisms to a varying degree; however, regardless of the planting type, the highest indices of forest stand influence were noted on the numbers of fungi, bacteria, and actinomycetes after soil afforestation with *Picea abies* L. According to Błońska [57], tree species affect soil pH and modify the conditions of organic matter degradation. The high C_mic_:C_org_ ratio in the soil [58] is indicative of the environment promoting the development of microorganisms. A year-long study by Walkiewicz et al. [15] has shown the highest C_mic_:C_org_ ratio in younger stands of coniferous and mixed forests, which is confirmed by our research wherein 19-year-old stands offered favorable conditions for the development of microorganisms. Plantings of common oak, *Larix decidua* M., and *Betula pendula* L. promoted the development of copiotrophic bacteria and actinomycetes. Thus, it can be assumed that a higher C_mic_:C_org_ ratio is typical of deciduous forests, which is consistent with the results of a study by Stolnikova et al. [59]. Moreover, the highest values of the ecophysiological diversity index (EP) determined for copiotrophic bacteria and fungi were found in the soil under the *Picea abies* L. plantings, while those for actinomycetes were found in the soil under the *Pinus sylvestris* L. plantings. The colony development index (CD) tested in the common oak stand had a weaker effect on the development of colonies of copiotrophic bacteria and actinomycetes, whereas CD determined in the *Picea abies* L. stand had a weaker effect on the development of fungi. Thus, the present study has shown that the structure of cultured copiotrophic bacteria, actinomycetes, and fungi changed under the influence of tree species composition. This change can be noticed in the proportion of k strategists to R strategists. The R strategy microorganisms are adapted to maximize the population growth rate, while those with the K strategy show a slow growth rate and are in turn adapted to maximize their competitive ability. The rapid development of microorganisms and the cooperation with soil fauna, deemed favorable due to the availability of nutrients, affects the degradation rate of plant litter and dead wood, with the latter representing a significant reservoir of carbon in forest systems [60]. Therefore, the specific responses of the microorganisms are probably due to the differences in carbon supply. According to Lu and Scheu [61] and Fanin et al. [62], the Gram+ bacteria are better at managing persistent carbon resources, while the Gram- ones are associated with slower growth rates. Coniferous forests with a low availability of labile carbon promote the development of oligotrophic microorganisms. These, in turn, are characterized by a low respiration rate and reduced biomass in the near-surface rhizosphere [61,62,63].

Due to the content of nutrients in the plant litter and plant root secretions, forest soils can modify the diversity of soil microorganisms and affect their survivability [26,56,64]. The differences in the structure of the communities colonizing the examined non-afforested soil *Picea abies* L., *Pinus sylvestris* L., *Larix decidua* M., and *Quercus robur* L. i *Betula pendula* L., and afforested soils, in our research were strongly related to the species composition of the stand, as evidenced by the OTU number of bacteria, which varied widely from 50,705 OTU in the soil from under the *Betula pendula* L. to 76,682 OTU in the soil from under the *Larix decidua* M. According to Ganault et al. [65], deciduous trees are more susceptible to microbial degradation than conifers. Mono-species coniferous stands are often considered to be microhabitats with a low concentration of nutrients or a high content of polyphenols or lignins in the environment [66]. The changes in the structure and diversity are influenced by the properties of the soil, the macrofauna communities, or tree crowns, which by modulating the availability of light have a significant impact on the condition of the ground cover and understory vegetation [56,64,65,66,67].

In turn, according to Norman and Barrett [68] and Baćmaga et al. [64], it is the pH that plays a major role in determining the structure of microbial communities in forest ecosystem soils. In the presented research, it significantly influenced the predominance of the bacterial phyla in the studied soils. *Actinobacteria* were found to be the major phylum in the non-afforested soil and in the soil under all trees, except for birch. They are associated with acidic, nutrient-poor, and often water-saturated (i.e., anaerobic) soils [56,69] and include aerophilic [70] and oligotrophic [71] groups. According to Shen et al. [72], acidic soils are usually richer in *Actinobacteria*, while soils with higher pH are mainly colonized by *Acidobacteria* [73] and *Proteobacteria* [72,74,75].

Our previous study addressing the changes in the composition of microbial communities of fresh coniferous forest, fresh mixed coniferous forest, fresh mixed forest, and moist mixed forest has shown that, apart from *Acidobacteria*, a high number of *Alphaproteobacteria* OTU were also determined in the soil despite its low pH (2.63–3.73). This suggests that the diversity of bacterial communities is not solely determined by soil pH but also by other environmental factors [56,66,76]. Furthermore, a fairly large number of *Proteobacteria* classes *Proteobacteria (α-Proteobacteria, β-Proteobacteria, γ-Proteobacteria*, and *δ-Proteobacteria)* may prefer different growth environments. In our research, the tree stand modified the structure of families. In the case of the soil from under the *Picea abies* L., the dominant families were *Gaiellaceae* phylum *Actinobacteria*, *Bradyrhizobiaceae* phylum *Firmicutes*, and *Ktedonobacteraceae* phylum *Chloroflexi*. Navarrete et al. [77] and Zhelezova et al. [78] have shown *Ktedonobacteraceae* (negatively correlated with organic matter content) to be the dominant family of bacteria in sandy soils. Rughöft et al. [79] identified this family of bacteria in savannah soils in the Kruger National Park in South Africa, while Li et al. [80] identified it in soils contaminated with copper. In the present research, the prevailing bacteria in the soil under *Pinus sylvestris* L. and *Larix decidua* M. were those of the *Gaiellaceae, Nocardioidaceae*, and *Hyphomicrobiaceae* families; in the soil under the oak, those from the *Gaiellaceae, Nocardioidaceae*, and *Rhodospirillaceae* families; and in the soils under the *Betula pendula* L., those from the *Burkholderiaceae, Rhodospirillaceae*, and *Gaiellaceae* families. Of the 12 genera classified into the phylum *Actinobacteria, Proteobacteria, Verrucomicrobia*, and *Firmicutes*, the *Rhodoplanes* had the largest share in the genus structure, followed by *Mycobacterium, Burkholderia*, and DA101. Moreover, all tree species increased the OTU number of the *Sphingomonas* and *Mycobacterium* genera, although to a different extent. *Larix decidua* M., *Pinus sylvestris* L., *Quercus robur* L., and *Picea abies* L. also increased the OTU number of the *Kribbella* genus. The bacteria of the genus *Rhodoplanes* and *Burkholderia* also dominated the bacterial community in the pine forest soils of the Manowo Forest District in northern Poland [81] and in the humid tropical forests of Costa Rica [82]. The genus *Rhodoplanes* was additionally found to prevail in the soils of the cleared forest in Indonesia [83] and, in our earlier research, in the forests of north-eastern Poland [57]. Moreover, Eaton et al. [82] have drawn attention to the bacteria of the *Solibacter, Comomonas, Azospirillum, Geobacter*, and *Bradyrhizobium* genera, whereas Lasota e al. [81] have drawn attention to those of the *Skermanella, Tsukamurella, Candidatus Solibacter*, and *Streptomyces* genera. When investigating forest and grass soils of Schwäbische Alb (Germany), Nacke et al. [84] pinpointed *Mycobacterium* as the most abundant genus of bacteria present in all soil samples and also highlighted *Amaricoccus* and *Methylocapsa* occurring in the soils of beech and spruce stands. In turn, Liu et al. [85] found that the soils of the deciduous and coniferous forests and meadows of the Songshan forest reserve in Yanqing (Beijing, China) were most abundant in the DA101 genus bacteria. Analyzing the results cited in the above literature [30,74,81,82,83,84] and the results of our study, it can be concluded that although the composition of bacterial communities in soils and their diversity vary, certain bacterial genera occur in most forest ecosystems, while others constitute the core microbiome of individual habitats. Thus, we have shown that the genus *Bacillus* is most characteristic for the spruce stand; *Sphingomonas* and *Iamia*, for the larch stand; and *Burkholderia*, for the birch stand. The diversity of bacteria in the soil from under different stands is due to their ecological adaptation to the prevailing habitat conditions.

As sensitive indicators of soil quality changes, the soil enzymes provide valuable information about the condition of the soil environment [2,31,32,33,43,81,86,87,88]. They mediate key soil processes and functions, such as organic matter degradation and nutrient cycle. The enhanced potential enzymatic activity in forest soils and their plant litter is attributed to extracellular enzymes that play important roles in biogeochemical cycles, catalyzing reactions related to organic matter degradation [89]. The activity of extracellular enzymes in litter degradation is mainly regulated by soil microorganisms supplying energy and nutrients for plant growth [90]. Our results confirmed both the higher number of microorganisms and the high activity of soil enzymes responsible for the C, N, and P cycle in the soil. According to the substrate stimulation model, the activity of soil enzymes responsible for the C cycle can be stimulated when the contents of their substrates (glucosides, disaccharides, and cellobioses) increase due to cellulose and hemicellulose degradation [89]. Microorganisms that are easily available and digestible by glucose may increase the content of β-1,4-glucosidase [90]. Our research results showed that all plantings stimulated activities of β-glucosidase and dehydrogenases. Being unable to accumulate in the extracellular environment [91], soil dehydrogenases can use both oxygen molecules as electron and proton acceptors and other compounds that occur in the cells of anaerobic microorganisms [92]. The activity of dehydrogenases is indicative of the presence of viable microbial cells [93,94]. In the present study, their high activity confirmed the high number of microorganisms in the soil from under all tree plantings. The enzymatic activity of the afforested soil was significantly higher than that of the non-afforested control soil. This is confirmed by the data on the positive impact of plant species on the soil microbiome [61,63,88,95] and the activity of enzymes [8,58,96,97] since the biochemical properties of soil are a derivative of plant species diversity and soil microbiome diversity [98,99,100].

## 5. Conclusions

The afforestation of post-arable soil with *Picea abies* L., *Pinus sylvestris* L., *Larix decidua* M., *Quercus robur* L., and *Betula pendula* L. had a beneficial, though varied, effect on the proliferation of soil microorganisms. Their strongest stimulating impact was observed on fungi and copiotrophic bacteria and a slightly lesser one on actinobacteria. The strongest growth promoter of microorganisms turned out to be *Quercus robur* L., followed by *Picea abies* L., whereas the weakest promoters appeared to be *Pinus sylvestris* L. and *Larix decidua* M. The soil sampled from the area afforested with *Quercus robur* L. also exhibited the highest enzymatic activity.

Post-arable soil afforestation modified the taxonomic structure; however, some regularities could be observed regardless of the effects of individual tree species. For instance, the OTU number of the *Sinobacteraceae* family bacteria decreased in all soil samples from the afforested areas. Furthermore, *Picea abies, Pinus sylvestris*, and *Quercus robur* L. increased the abundance of *Bradyrhizobiaceae*, whereas *Quercus robur* L., *Larix decidua M.*, and *Pinus sylvestris* L. decreased the abundance of *Nocardioidaceae*.

The genus *Bacillus* turned out to be the most typical genus found in the soil from the area afforested with *Picea abies* L., whereas *Pseudonocardia* did in that area afforested with *Pinus sylvestris* L.; *Iamia* did in that afforested with *Larix decidua* M.; and *Burkholderia* did in that afforested with *Betula pendula* L. The core genus prevailing in the soil samples from the plots afforested with the three species tested turned out to be *Rhodoplanes*.

## Figures and Tables

**Table 1:** The Shannon–Weaver (H’) indexes of microbial diversity (greater than or equal to 1%).

Taxa	C	Pa	Ps	Ld	Qr	Bp
Phylum	1.86 ^a^	1.86 ^a^	1.62 ^b^	1.61 ^b^	1.67 ^b^	1.82 ^a^
Class	2.72 ^a^	2.47 ^b^	2.50 ^b^	2.50 ^b^	2.54 ^b^	2.67 ^a^
Order	2.90 ^a^	2.70 ^c^	2.62 ^c^	2.68 ^c^	2.78 ^b^	2.93 ^a^
Family	3.08 ^b^	3.05 ^b^	2.99 ^b^	3.05 ^b^	3.17 ^a^	3.02 ^b^
Genus	2.05 ^d^	2.16 ^c^	2.32 ^a^	2.37 ^a^	2.25 ^b^	1.68 ^e^

C—non-afforested land, Pa—*Picea abies* L., Ps—*Pinus sylvestris* L., Ld—*Larix decidua* M., Qr—*Quercus robur* L., and Bp—*Betula pendula* L. Homogeneous groups (denoted with letters a–e) were calculated separately for each taxa.

## Data Availability

Sequencing data have been deposited with GenBank NCBI. They are available online: https://www.ncbi.nlm.nih.gov/nuccore/?term=MW601841:MW601938[accn] (accessed on 21 February 2021) under accession numbers MW601841–MW601938.

## Supplementary Materials

The following supporting information can be downloaded at: https://www.mdpi.com/article/10.3390/ma15093287/s1, Table S1: the granulometric composition of soils; Table S2: the physicochemical properties of soils; Table S3: the characteristics of soil; and Table S4: the enzymatic activity in soil, kg^−1^ DM of soil h^−1^.
