# Supplementary material for: Bacteria and Soil Enzymes Supporting the Valorization of Forested Soils
Source: Materials (Basel). 2022 May 4;15(9):3287. doi: 10.3390/ma15093287 (PMC9102912; doi:10.3390/ma15093287)
Supplement: Supplementary file 1 [file materials-15-03287-s001.zip › materials-1671846-supplementary.pdf]

materials

Supplementary Materials

Bacteria and Soil Enzymes Supporting the Valorization of Forested Soils

Table S1. Granulometric composition of soils.

Object	% of fractions (*d*, mm)			Kind of soil
	sand<br>2.00 ≥ *d* > 0.05	silt<br>0.05 ≥ *d* > 0.002	clay<br>*d* ≤ 0.002	
Non-afforested land	84.43[a]	14.22[b]	1.35[b]	Sandy loam (SL)
Picea abies L.	84.83[a]	14.16[b]	1.01[e]	Sandy loam (SL)
Pinus sylvestris L.	86.15[a]	12.63[d]	1.22[c]	Sandy loam (SL)
Larix decidua M.	85.85[a]	13.12[c]	1.03[e]	Sandy loam (SL)
Quercus robur L.	81.95[b]	16.38[a]	1.67[a]	Sandy loam (SL)
Betula pendula L.	86.56[a]	13.33[c]	1.11[d]	Sandy loam (SL)
$LSD_{0.05}$	2.281	0.377	0.034	-

LSD – Least Significant Difference. Homogeneous groups denoted with letters (a-e) were calculated separately for each enzyme. LSD – Least Significant Difference

Table S2. Physicochemical properties of soils.

Object	pH_{KCl}	HAC	TEB	CEC	BS [%]
		mmol(+) kg^{-1}			
Non-afforested land	4.90[c]	31.6[c]	52.4[d]	84.0[c]	62.4[c]
Picea abies L.	4.40[e]	40.5[a]	52.3[d]	92.8[a]	56.4[e]
Pinus sylvestris L.	5.60[b]	27.3[e]	54.4[c]	81.7[cd]	66.4[b]
Larix decidua M.	5.90[a]	23.6[f]	58.0[a]	81.5[d]	71.1[a]
Quercus robur L.	5.50[b]	30.2[d]	56.2[b]	86.4[b]	65.0[b]
Betula pendula L.	4.70[d]	32.5[b]	47.2[e]	79.7[e]	59.2[d]
$LSD_{0.05}$	0.139	0.842	1.437	2.268	1.708

HAC - hydrolytic acidity; EBC - exchangeable base cations; CEC - cation exchange capacity; BS - base saturation. LSD – Least Significant Difference. Homogeneous groups denoted with letters (a-e) were calculated separately for each enzyme. LSD – Least Significant Difference

Table S3. Characteristics of soil.

**Object**	**Content in 1 kg DM**								
	**Total, g**		**Available, mg**			**Exchangeable, mg**			
	**$C_{organic}$**	**N**	**P**	**K**	**Mg**	**K**	**Ca**	**Na**	**Mg**
Non-afforested land	5.50^{e}	0.66^{d}	29.9^{e}	85.5^{c}	20.0^{f}	134.0^{c}	214.3^{d}	20.0^{d}	18.0^{e}
Picea abies L.	17.3^{a}	0.91^{a}	9.98^{d}	37.3^{f}	36.0^{b}	80.0^{e}	325.0^{c}	40.0^{c}	34.0^{c}
Pinus sylvestris L.	6.90^{d}	0.62^{e}	49.0^{c}	49.8^{e}	32.0^{d}	82.0^{e}	400.0^{b}	20.0^{d}	25.0^{d}
Larix decidua M.	9.40^{c}	0.67^{d}	69.9^{a}	107.9^{a}	34.0^{c}	144.0^{b}	500.0^{a}	60.0^{b}	42.0^{b}
Quercus robur L.	13.5^{b}	0.73^{b}	43.0^{d}	103.7^{b}	50.0^{a}	164.0^{a}	325.0^{c}	20.0^{d}	54.8^{a}
Betula pendula L.	9.30^{c}	0.71^{c}	67.7^{b}	70.5^{d}	28.0^{e}	128.0^{d}	214.3^{d}	80.0^{a}	25.0^{d}
$LSD_{0.05}$	0.297	0.019	1.329	2.152	0.927	3.380	9.255	1.240	0.949

LSD – Least Significant Difference. Homogeneous groups denoted with letters (a-e) were calculated separately for each enzyme. LSD – Least Significant Difference

Table S4. Enzymatic activity in soil, kg^{-1} DM of soil $h^{-1.}$

**Object**	**Deh (µMol TFF)**	**Cat (Mol O_2)**	**Ure (mMol N-NH_4)**	**Pac**	**Pal**	**Glu**	**Aryl**
				**(mMol 4-nitrophenol PN)**			
Non-afforested land	9.886^{e}	3.072^{e}	1.044^{cd}	2.151^{c}	0.237^{e}	0.354^{f}	0.190^{e}
Picea abies L.	25.96^{b}	3.218^{d}	0.843^{e}	2.434^{b}	0.278^{d}	0.831^{b}	0.371^{c}
Pinus sylvestris L.	21.95^{c}	3.355^{a}	1.082^{c}	2.074^{c}	0.506^{b}	0.613^{c}	0.334^{d}
Larix decidua M.	21.40^{c}	3.353^{a}	1.554^{a}	1.783^{d}	0.906^{a}	0.465^{e}	0.417^{b}
Quercus robur L.	43.51^{a}	3.288^{b}	1.192^{b}	3.455^{a}	0.417^{c}	1.251^{a}	0.523^{a}
Betula pendula L.	14.10^{d}	3.235^{c}	1.020^{d}	2.170^{c}	0.239^{e}	0.555^{d}	0.328^{d}
$LSD_{0.05}$	0.867	0.011	0.58	0.129	0.020	0.018	0.019

Deh - dehydrogenases; Ure - urease, Pal – alkaline phosphatase, Pac – acid phosphatase, Aryl - arylsulphatase, Glu – *β*-glucosidase. Homogeneous groups denoted with letters (a-e) were calculated separately for each enzyme. LSD – Least Significant Difference.
